# The release of sexual conflict after sex loss is associated with evolutionary changes in gene expression

**DOI:** 10.1098/rspb.2024.2631

**Published:** 2025-01-29

**Authors:** Hélène Defendini, Nathalie Prunier-Leterme, Stéphanie Robin, Sonia Lameiras, Sylvain Baulande, Jean-Christophe Simon, Julie Jaquiéry

**Affiliations:** ^1^ UMR 1349, IGEPP, INRAE, Institut Agro, Université de Rennes, 35653 Le Rheu and 35000 Rennes, France; ^2^ Institut Curie, PSL University, ICGex Next-Generation Sequencing Platform, Paris 75005, France

**Keywords:** sexual antagonism, parthenogenesis, asexuality, transcriptomics, sex-biased gene expression, sexual dimorphism

## Abstract

Sexual conflict can arise because males and females, while sharing most of their genome, can have different phenotypic optima. Sexually dimorphic gene expression may help reduce conflict, but the expression of many genes may remain sub-optimal owing to unresolved tensions between the sexes. Asexual lineages lack such conflict, making them relevant models for understanding the extent to which sexual conflict influences gene expression. We investigate the evolution of sexual conflict subsequent to sex loss by contrasting the gene expression patterns of sexual and asexual lineages in the pea aphid *Acyrthosiphon pisum*. Although asexual lineages of this aphid produce a small number of males in autumn, their mating opportunities are limited because of geographic isolation between sexual and asexual lineages. Therefore, gene expression in parthenogenetic females of asexual lineages is no longer constrained by that of other morphs. We found that the expression of genes in males from asexual lineages tended towards the parthenogenetic female optimum, in agreement with theoretical predictions. Surprisingly, males and parthenogenetic females of asexual lineages overexpressed genes normally found in the ovaries and testes of sexual morphs. These changes in gene expression in asexual lineages may arise from the relaxation of selection or the dysregulation of gene networks otherwise used in sexual lineages.

## 1. Introduction

Males and females share most of their genome, but the sexes may have different fitness optima for many phenotypic traits. This can lead to inter-locus sexual conflict if different genes are involved depending on sex. If the genetic architecture of traits is shared between the sexes, this creates intra-locus sexual conflict, where a trait is prevented from evolving towards its fitness optimum in males, females or both [1,2]. The evolution of sexually dimorphic gene expression may help to reduce conflict, although the expression of many genes may still be sub-optimal owing to remaining genetic and functional constraints [3–5]. As a result, genes with a current sexually dimorphic expression should reflect different stages of resolved or partially unresolved sexual conflicts [6]. The extent to which these conflicts are resolved in natural populations remains difficult to assess because of the small effects of the presumably numerous sexually antagonistic loci distributed throughout the genome [6–8].

One way of addressing this question is to study the evolution of the transcriptome in sexual species when selective pressures are artificially altered. Enforcing monogamy in polygamous species is expected to reduce sexual selection on males and thus relax sexually antagonistic selection [9]. Evolution under monogamy would thus lead to a change in sex-biased gene expression in both sexes towards the female optimum, resulting in increased expression of female-biased genes and decreased expression of male-biased genes. Three studies that experimentally evolved different polygamous *Drosophila* species under monogamy for more than 100 generations had mixed results. An initial study in *D. melanogaster* showed a pattern of feminization of the male and female transcriptomes [10], but these results were not confirmed in a second study [11]. In a third study on *D. pseudoobscura*, shifts in the expression of sex-biased genes were mainly tissue- and condition-dependent and, contrary to predictions, transcriptomes tended to be masculinized [12]. Major limitations of these studies are that evolutionary time elapsed since the change in mating system is short and that enforcing monogamy does not completely remove selection on males because their genes are still passed on to the next generation.

Species that have lost sex are of particular interest because conflict between sexes no longer applies. The comparison of the transcriptomes of asexual and closely related sexual species provides a good opportunity to study gene expression evolution after the suppression of any form of sexual antagonism following sex loss. Any asexual lineage that invests more in female functions compared to sexual species–at the expense of males that are absent and thus do not constrain gene expression in females–should be favoured by selection. Thus, we expect higher expression of female-biased genes and reduced expression of male-biased genes in asexual as compared to sexual species [13,14].

Parker *et al*. [13] compared expression of sex-biased genes in five pairs of sexual and closely related asexual species of *Timema* stick insects. Contrary to theoretical predictions, they found evidence for an overall masculinization of sex-biased gene expression in asexual females and hypothesized that this is owing to shifts in female trait optima levels following the loss of sex. Indeed, for cases where an obligate sexual species loses sexual reproduction, not only does the intensity of conflict change, but so does the optimal expression of the new asexual morph, which can differ from that of sexual females. For instance, the newly evolved asexual morph no longer needs to attract and mate with males, and has no more contact with seminal fluids, which could lead to a significant shift in phenotypic optima. Huylmans *et al*. [14] also compared gene expression patterns in sexual and asexual species of the brine shrimp *Artemia*. They did not show particular shifts and explained their results by the fast turnover of sex-biased genes, which had high rates of expression divergence even within sexual *Artemia* species.

A point not addressed in the previous studies [13,14] is that, in addition to the suppression of sexual conflict, asexuality is associated with a lack of genome recombination. This may affect the evolution of gene expression in several ways. The absence of recombination impedes the efficacy of selection to act upon asexual genomes and prevents the purging of deleterious mutations [15]. Hence, gene expression is likely to evolve towards optimal levels at a slower rate in asexual lineages. Lack of recombination also leads to independent evolution of asexual lineages that should enhance expression divergence among asexual lineages in the long term. Thus, global gene expression should be more divergent and variable between asexual lineages than between sexual lineages, especially if asexual lineages have diverged long time ago.

In this context, aphids, which evolved partial asexuality long ago and have secondarily lost the sexual phase multiple times, are of particular interest. The common sexual ancestor of aphids acquired the ability to alternate between parthenogenetic and sexual reproduction *ca* 250 Myr ago [16,17]. This alternation of many asexual (apomictic parthenogenesis) generations and one sexual generation a year is called cyclical parthenogenesis (CP) and involves three distinct reproductive morphs: viviparous parthenogenetic females, which are present for the main part of the aphid life cycle (*ca* 10–15 generations per year), males and oviparous sexual females, both of which are produced once a year. Importantly, parthenogenetic females are exclusively viviparous, never mate, have no sexual interaction with males and never produce eggs. In contrast, the sexual female morph (generated only under specific photoperiodic conditions in autumn) reproduces exclusively sexually and produces only eggs. As a result, the two female morphs are phenotypically distinct and cannot switch from sexual to parthenogenetic reproduction and *vice versa*. In aphids, sex is determined chromosomally by an XX/X0 system, with X0 males produced by the random elimination of an X chromosome from the germ line [18] and XX sexual and parthenogenetic females that are genetically identical to their parthenogenetic mother. The single X in males is dosage compensated at the whole-body scale, but more surprisingly also within the adult gonads [19,20], perhaps because spermatogenesis is already complete at the adult stage [21]. Interestingly, previous work has shown that sexual conflicts may be of importance in CP aphids [20]. Mathematical modelling showed that the conditions of invasion of sexually antagonistic non-synonymous mutations favourable to males are less restrictive on the X than on autosomes (and *vice versa* for male deleterious alleles), making the X chromosome more favourable for males [20]. If the evolution of sex-biased expression can alleviate sexual conflict, the X should be enriched in male-biased genes and this is indeed what is observed [19,20]. The agreement between theoretical predictions and empirical data suggests that sexual conflict is an important player in genome evolution and gene expression in aphids.

Remarkably, some CP aphid lineages have secondarily lost the ability to reproduce sexually, becoming obligate parthenogens (OP). OP lineages produce only parthenogenetic females and sometimes males, but no sexual females. Since the egg resulting from sexual reproduction is the sole cold-resistant stage in aphids, only CP lineages can survive in cold-winter regions [22,23]. Contrastingly, OP lineages cannot produce eggs (owing to the absence of sexual females); they are thus frost sensitive and are found in mild-winter regions where their ability to maintain parthenogenetic development in winter gives them a demographic advantage over CP lineages [22]. This geographic separation of CP and OP lineages owing to climate has been shown for several aphid species [24–26]. As a result, OP males have low opportunity to find mating partners (i.e. sexual females from CP lineages) and to pass on their genes to the next generation. These OP males are supposed to be under relaxed selection, and some of their traits do indeed show evidence of a slight decay compared with CP males [26]. Based on this knowledge, we hypothesized that in OP lineages, only one morph (the parthenogenetic female) is under strong selection; hence, conflicts between morphs are relaxed. This may affect the evolution of their transcriptome—in particular, morph-biased genes that may have been involved in an intra-locus conflict that no longer exists in OP lineages. As gene expression in OP parthenogenetic females is no longer constrained by expression in other morphs, we suppose that OP lineages that overexpress parthenogenetic female-biased genes would be favoured by selection. Since the maintenance of differential gene expression between sexes when intra-locus conflict has disappeared might no longer benefit OP lineages, we also expect selection to favour OP lineages whose male gene expression evolves towards parthenogenetic female optimum. Importantly, the parthenogenetic female morph already exists in CP populations and is the main morph, so that we do not expect changes in OP parthenogenetic female fitness optima. Aphids thus contrast with other asexual systems (as *Timema* or *Artemia* [13,14]) in which sexual females turn to asexual females following sex loss (hence with possible new fitness optima).

To investigate how loss of sex alters gene expression patterns, we performed whole-body transcriptome sequencing (RNA-seq) on parthenogenetic females and males in four OP lineages and on parthenogenetic females, males and sexual females in four CP lineages of the pea aphid *Acyrthosiphon pisum*. First, we aimed to evaluate whether the lack of recombination in OP lineages increases gene expression differentiation between OP lineages since genomes of asexual species are expected to evolve independently without mixing since sex loss. For this purpose, we examined the genetic relationships between OP and CP lineages and tested whether gene expression was more divergent between OP lineages than between CP lineages. Second, to test the hypothesis that the suppression of sexual conflict in OP lineages could result in a change in gene expression patterns, we first identified morph-biased genes using independent RNA-seq data and then tested for differences in gene expression between CP and OP parthenogenetic females and CP and OP males for different categories of genes. To better understand the observed changes in gene expression between OP and CP parthenogenetic females or males, we subdivided these morph-biased genes according to their chromosomal location (X vs autosomes), the degree of expression bias and the tissue in which they were most expressed.

## 2. Material and methods

### (a) Biological samples

Eight genetically distinct lineages of *Acyrthosiphon pisum* were selected from a large panel of lineages used in another study [26] that were collected from clover fields in France and screened for their ability to produce sexual morphs. Four of them, characterized as OP lineages, were collected in the west of France and the four others collected in the east were characterized as CP lineages. These eight lineages were selected from those that produced enough males to provide the necessary biological material, while being distributed throughout the phylogenetic tree in [26].

To produce sexual morphs, the eight lineages were reared under sex-inducing conditions [27]. Briefly, one 1-day-old larva was placed on a *Vicia faba* plant stored at 18°C with a 16 : 8 light : dark (L:D) photoperiod regime for 1 week. Then, the 1-week-old individual was moved to sex-inducing short-day conditions (12 : 12 L:D regime at 18°C). Six days later, four 1-day-old larvae (i.e. the second generation) were placed on a new plant and maintained under short-day conditions. Once adults, the four individuals produced sexual morphs (males and oviparous sexual females in CP lineages, males only in OP lineages). These four individuals were transferred to a new plant every day in order to avoid crowding and to precisely record the age of the future sexuals. The plants hosting the future sexuals were immediately returned to a 16 : 8 L:D photoperiod. We isolated sexual females from males prior to sexual maturation (at the fourth larval stage) to ensure that sexual morphs were virgin. We collected adult individuals 2 to 3 days after imaginal moulting. Parthenogenetic females were obtained by rearing the eight lineages under a 16 : 8 L:D photoperiod regime at 18°C. One 1-day-old larva of each lineage was deposited on a *V. faba* plant. Once adult, females start to lay offspring. We waited for six offspring to be laid, which takes 1 day, and the female was then removed from the plant. These individuals were collected 2–3 days after their imaginal moult. All individuals were flash-frozen in liquid nitrogen and stored at −80°C. We pooled 6–10 males per replicate, three sexual females per replicate and two parthenogenetic females per replicate. Two biological replicates per morph and lineage were generated. We recognize that the use of different photoperiodic regimes–necessary to obtain the different morphs–could affect gene expression, but this effect was minimized by reducing the time spent in 12 : 12 L:D as much as possible.

### (b) RNA sequencing

We performed a total of 40 RNA extractions (4 CP lineages × 3 morphs × 2 replicates and 4 OP lineages × 2 morphs × 2 replicates) using the SV Total RNA Isolation System (Promega) following manufacturer’s instructions. The quality of the RNA samples was checked on Bioanalyzer (Agilent) and quantified on Nanodrop (Thermo Scientific). Libraries were prepared from 500 ng of total RNA using the Illumina® Stranded mRNA Prep Ligation library preparation kit, which allows to perform a strand-specific sequencing. This protocol includes a first step of polyA selection using magnetic beads. After RNA fragmentation, cDNA synthesis was performed and resulting fragments were used for dA-tailing followed by ligation of RNA Index Anchors. PCR amplification with indexed primers (IDT for Illumina RNA UD Indexes) was finally achieved, to generate the final cDNA libraries. Library quantification and quality assessment were performed using Qubit fluorometric assay (Invitrogen) with dsDNA HS (High Sensitivity) Assay Kit and LabChip GX Touch using a High Sensitivity DNA chip (Perkin Elmer). Libraries were then equimolarly pooled and quantified by qPCR using the KAPA library quantification kit (Roche). Sequencing was carried out on the NovaSeq 6000 instrument from Illumina using paired-end 2 × 100 bp, to obtain around 40 million raw paired-end reads per sample (electronic supplementary material, table S1).

Processing of the RNA-seq data was carried out using the nf-core RNA-seq pipeline v.3.4 (https://nf-co.re/rnaseq/3.4). This pipeline was based on Nextflow v.21.04.0, using the default settings and adding the option --remove_ribo_rna. Reads were mapped to the reference genome *Acyrthosiphon pisum* JIC1 v.1.0 [28] available in AphidBase [29] with STAR v.2.6.1d [30]. Transcript quantification was performed with featureCounts v.1.6.0 [31] according to the *A. pisum* JIC1 v.1.0 gene annotation with default settings except -C -p -s 2 M --fraction. We then used the R package edgeR v.3.38.4 [32] to normalize the libraries (trimmed mean of M values (TMM) method, *p* = 0.75) and calculate count per million (CPM) on R v.4.3.1 [33]. Only genes with normalized CPM > 1 in at least eight libraries (out of the 40 libraries), i.e. 14 319 genes, were retained in the analyses. The CPM per gene of the two replicates of the same condition was averaged unless otherwise stated.

### (c) Genetic relationships between OP and CP lineages

To gain insight into the genetic relationships between the eight pea aphid lineages, we built a tree using single nucleotide polymorphism (SNP) variants retrieved from the transcriptomic data. SNP variants with a genotype quality above 20 were identified and further filtered for minimum depth of 80 in each library with bcftools and vcftools [34]. We then built a neighbour-joining tree using Nei distances [35] calculated from 11 449 high-quality SNPs using the *nei.dist* function from poppr R package [36]. A total of 1000 bootstraps were performed using the *aboot* function from the same package. One parthenogenetic female library per lineage was used to build the tree (electronic supplementary material S1A). A principal component analysis (PCA) was also carried out on these eight lineages with the *dudi.pca* function from the ade4 R package [37].

### (d) Comparison of the divergence in gene expression between CP and OP lineages

The possible effect of the lack of genome recombination on gene expression evolution was tested by comparing transcriptomic distances in CP and in OP lineages. Transcriptomic Euclidean distances between samples were estimated using the *dist* R function after a normalization step by gene on CPM. We first tested for a correlation between the Nei genetic distance and the male (or female) transcriptomic distance matrices using Mantel tests implemented in the R package vegan [38]. We then measured the average distance between a focal lineage and the three other lineages with the same reproductive mode and tested whether distances differed between reproductive modes using two-sided Mann-Whitney tests. Such tests were performed on genetic distances, transcriptomic distances (measured per sex) and transcriptomic distances corrected for genetic distances (i.e. the ratio of transcriptomic to genetic distances).

### (e) Change in morph-biased and unbiased gene expression between OP and CP lineages

To investigate how gene expression may change between OP and CP lineages, we first computed—for each gene—the average CPM over the four parthenogenetic females from OP lineages (replicates from the same lineage were already averaged) and over the four parthenogenetic females from CP lineages. The log_2_ of the OP to CP ratio of these values (adding 0.05 to the denominator and numerator to avoid division by zero) was then calculated. Genes for which the ratio is greater than zero are expressed more by OP than by CP parthenogenetic females and *vice versa*. The same approach was applied to males. We then separated these genes into different expression classes (which were defined using independent RNA-seq data from an *A. pisum* outgroup from the alfalfa host race [19,20]—see the next paragraph), first according to their morph-biased expression status (in males, parthenogenetic females and sexual females in the alfalfa outgroup) and the degree of bias, then as a function of their tissue-biased expression status (in head, gonads and legs) and finally by their chromosomal localization (X vs autosomes). For each of these classes of genes, we tested for a deviation of the OP-to-CP log_2_ ratio from zero to determine whether some specific gene classes showed change in expression between OP and CP lineages. For this, we used a Wilcoxon signed-rank test with continuity correction implemented in the *wilcox.test* R function. The rationale behind these analyses was to evidence some general but probably weak trends on some gene categories (e.g. sex-biased genes) that would probably be difficult to detect at the level of individual genes.

To identify morph-biased and morph-limited gene classes for the above analyses without relying on our main focal RNA-seq data from the clover host race (which could lead to spurious conclusions if the same dataset is used as explanatory and response variable), we used published RNA-seq data of an *A. pisum* outgroup from the alfalfa host race [20]. The dataset consisted of whole-body RNA-seq data with two replicates for each of the three morphs (male, sexual female and parthenogenetic female). Sexual individuals were virgin. RNA-seq data were processed with the same parameters as above and only genes with CPM > 1 in at least two libraries (out of the six libraries) were retained. Genes showing a morph-biased or morph-limited expression were identified the following way: differential expression analysis among the three morphs was conducted using edgeR v.4.2.1 [32], applying genewise exact tests for differences in means between two groups (assuming a negative binomial distribution of counts). *P*-values were adjusted for multiple comparisons using the Benjamini–Hochberg method [39]. Three pairwise comparisons were made for each gene: male vs parthenogenetic female, sexual female vs parthenogenetic female and sexual female vs male. Genes consistently overexpressed by one morph in both of the comparisons with the two other morphs (with adjusted *p*-values < 0.05 and absolute log_2_ fold-change > 1) were classified as morph-biased (either male-biased, sexual female-biased or parthenogenetic female-biased) and as morph-limited if the absolute log_2_ fold-change was > 2. Genes that were neither morph-biased nor morph-limited were defined as unbiased. Second, to further refine these categories of gene expression with tissue bias information, we used another RNA-seq dataset of the same *A. pisum* alfalfa outgroup in which three tissues (head, legs and gonads) were investigated in the three morphs [19]. Sexual individuals were also virgin. Genes with normalized CPM > 1 in at least two libraries (out of the 18 libraries) were retained in the analyses. We then identified genes predominantly expressed in a single tissue (head, legs or gonads) of a single morph (male, sexual female or parthenogenetic female) by performing genewise exact tests with edgeR, as above. Genes consistently overexpressed in one tissue in both comparisons with the other two tissues from the same morph (with adjusted *p*-values < 0.05 and absolute log_2_ fold-change > 1) were classified as tissue-biased (i.e. head-, gonad- or leg-biased) in that morph. X-linked and autosomal genes were identified based on the chromosome-scale genome assembly of *A. pisum* (JIC1 [28]). Finally, we also wanted to check that the results were robust regardless of how the differentially expressed (DE) genes were determined. Instead of using RNAseq data from the alfalfa outgroup, we used the RNAseq data from one of the CP clover lineage to categorize genes as DE (using same methods as described above). To avoid circularity (resulting from including the same data as explanatory and response variables), we recalculated the OP-to-CP ratio but without the focal CP clover lineage (i.e. using only the three other CP lineages and the four OP lineages) and plotted it for the different gene expression classes. We repeated this operation three times, using a different focal CP clover lineage. These results are presented in electronic supplementary material S2. Reassuringly, most morph-biased genes identified using the *A. pisum* alfalfa outgroup were also morph-biased in CP lineages in the clover host race (electronic supplementary material S3).

Further analyses were conducted. First, to investigate how the OP and CP samples grouped, we performed PCAs on the 40 RNAseq libraries (considering all genes or separating them by expression class as defined with the alfalfa outgroup). Then, without focusing on specific gene categories, we identified DE genes between OP and CP lineages separately for males and parthenogenetic females with GlmmTMB [40] and performed GO enrichment tests. We also tested the hypothesis that the transcriptomes of parthenogenetic females and males of the same lineage should be more correlated in OP lineages (because selection is expected to act only on parthenogenetic females) than in CP lineages (where selection could act in different directions on parthenogenetic females and males). Finally, we verified that dosage compensation (already demonstrated in CP lineages [19,20]) operates similarly in OP lineages. As all these analyses presented limited additional findings, the corresponding methods and results are provided solely as supplementary material (electronic supplementary materials S4 and S5, suppl. text S1, tables S2 and S3).

## 3. Results

### (a) Genetic relationships between OP and CP lineages

The neighbour-joining tree built with the 11 449 SNPs from the transcriptomic data revealed that the eight OP and CP lineages grouped by reproductive mode (electronic supplementary material S1A), with good bootstrap support. The first axis of the PCA also separated lineages by their mode of reproduction, which explained 18.5% of the variance (electronic supplementary material S1B). One OP lineage (OP1) was closer to CP lineages both on the tree and on the PCA. Although mean Nei genetic distances between OP lineages (0.157) were slightly but significantly lower than those measured between CP lineages (0.163, *p* = 0.029, Mann-Whitney test, electronic supplementary material, table S4), we did not observe any shallower branching between OP lineages in the neighbour-joining tree (electronic supplementary material S1A). These results indicate that it is unlikely that the four OP lineages studied here have a very recent common ancestor.

### (b) No difference in expression divergence between OP and between CP lineages

We evaluated the hypothesis that the absence of recombination would result in higher expression divergence within OP lineages than CP lineages using transcriptomic distances. Parthenogenetic females showed the same average level of divergence regardless of reproductive mode (OP: 138.5, CP: 140.5, *p* = 0.88, Mann-Whitney test, electronic supplementary material, table S4) as males (OP: 137.0, CP: 144.3, *p* = 0.20, electronic supplementary material, table S4). As Nei genetic distances were significantly lower within OP lineages compared to CP lineages (see above) and because transcriptomic distances increased significantly with genetic distances for both parthenogenetic females (Spearman correlation coefficient *r_s_* = 0.489, *p* = 0.009, Mantel test) and males (*r_s_* = 0.479, *p* = 0.013), transcriptomic distances were corrected for genetic distances. We did not observe differences for parthenogenetic females (OP: 885.4, CP: 862.7, *p* = 0.11, Mann-Whitney test) or males (OP: 877.0, CP: 885.9, *p* = 0.89).

### (c) OP parthenogenetic females overexpress sexual female-biased genes

**Figure 1.**
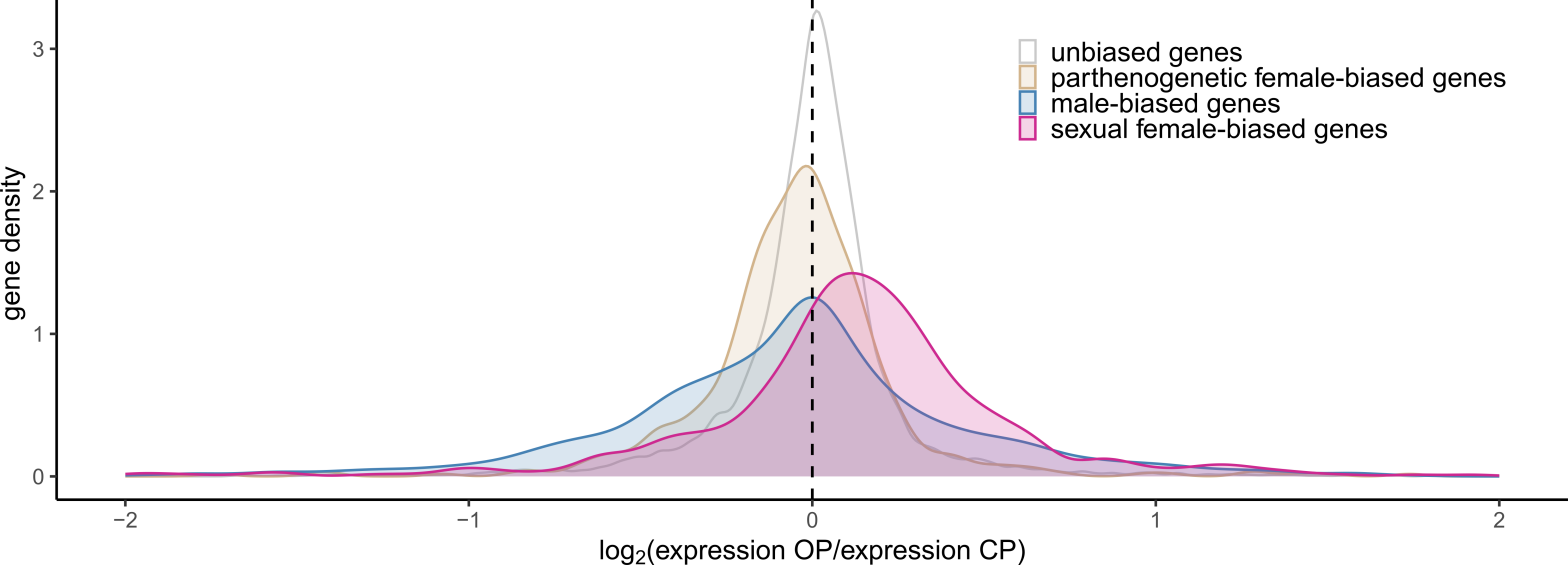
Gene expression changes between parthenogenetic females from OP and CP lineages of the pea aphid for different categories of genes (in different colours). The OP-to-CP log_2_-ratio of gene expression (in CPM) is shown on the *x*-axis. Colours discriminate between sexual female-biased genes (pink), parthenogenetic female-biased genes (beige), male-biased genes (blue) and unbiased genes (white). These different gene expression classes were determined using RNAseq data from an independent dataset (the alfalfa outgroup).

**Figure 2.**
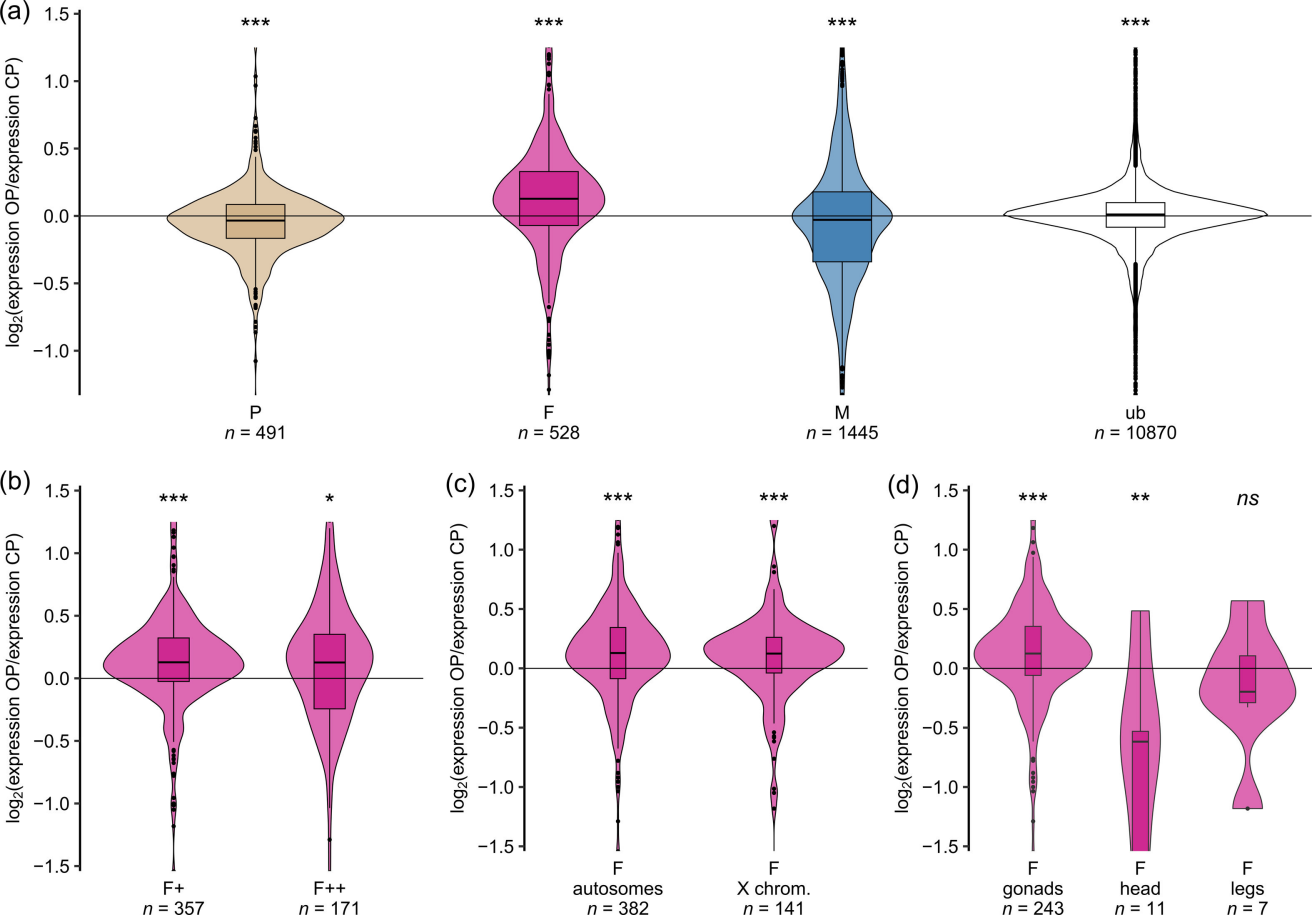
Log_2_-ratio of OP-to-CP gene expression in parthenogenetic females of the pea aphid for different categories of genes. OP-to-CP expression ratio as a function of: (*a*) morph-biased status of genes, (*b*) degree of morph bias (+: biased only or ++: limited), (*c*) chromosomal location of genes and (*d*) morph- and tissue-biased status of genes. Colours discriminate between sexual female-biased genes (F, in pink), parthenogenetic female-biased genes (P, in beige), male-biased genes (M, in blue) and unbiased genes (ub, in white). These different gene expression classes were determined using RNAseq data from an independent dataset (the alfalfa outgroup). The significance level of Wilcoxon signed-rank tests against 0 is indicated by asterisks (*** < 0.001, ** < 0.01, * < 0.05, *ns*. ≥ 0.05).

OP parthenogenetic females expressed sexual female-biased genes at a higher level than CP parthenogenetic females (figure 1), with a median OP-to-CP log_2_-ratio (hereafter referred to as *Mdn*) of 0.128 (Wilcoxon signed-rank test against 0, *p* < 10^–12^). Remarkably*,* this pattern was consistent for all OP lineages when pairwise comparisons were made (electronic supplementary material S6) and was also robust to the way the different gene expression classes were identified (i.e. using the alfalfa outgroup vs clover; electronic supplementary material S2). In contrast, no substantial shift was observed for the other classes of genes (i.e. male-biased, parthenogenetic female-biased and unbiased genes), although the effect was significant in each case owing to large sample size (figures 1 and 2*a*). The unbiased genes differed from the morph-biased genes by their much lower variance, indicating higher stability of expression.

To test whether a specific category of genes was responsible for the unexpected increased expression of sexual female-biased genes in OP parthenogenetic females, we first separated these genes by the degree of their morph bias in the alfalfa outgroup: both sexual female-biased genes (i.e. F+; *Mdn* = 0.128, *p* < 10^–13^) and sexual female-limited genes (i.e. F++; *Mdn* = 0.126*, p* = 0.038) contributed to the high OP-to-CP expression ratio (figure 2*b*). We then looked for an effect of the chromosome type: both sexual female-biased genes assigned to autosomes (*Mdn* = 0.128, *p* < 10^–8^) and to the X chromosome (*Mdn* = 0.124, *p* < 10^–4^) showed a high OP-to-CP expression ratio (figure 2*c*). Finally, the sub-classification of sexual female-biased genes according to their expression in different tissues showed that genes more expressed in sexual female ovaries contributed the most to the high OP-to-CP expression ratio (*Mdn* = 0.124, *p* < 10^–6^, figure 2*d*). For comparison, the same type of analyses were performed for male-biased and parthenogenetic female-biased genes, but no strong trend was found (see electronic supplementary material S7A–F). Analyses by chromosome type can be found in electronic supplementary materials S8 and S9. In summary, parthenogenetic females from OP lineages unexpectedly overexpress sexual female-biased genes that are mainly expressed in female ovaries, and, contrary to expectations, they do not overexpress parthenogenetic female-biased genes.

### (d) OP males overexpress parthenogenetic female-biased but also male-biased genes

**Figure 3.**
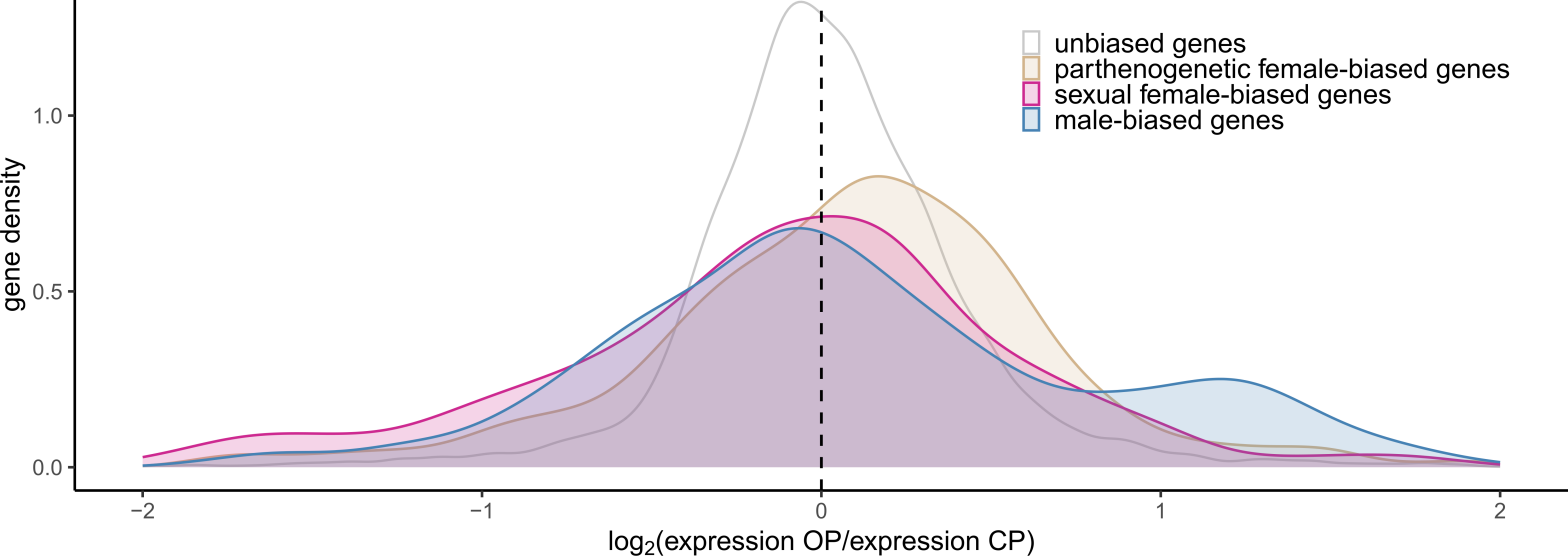
Gene expression changes between males from OP and CP lineages of the pea aphid for different categories of genes (in different colours, same as figure 1).

**Figure 4.**
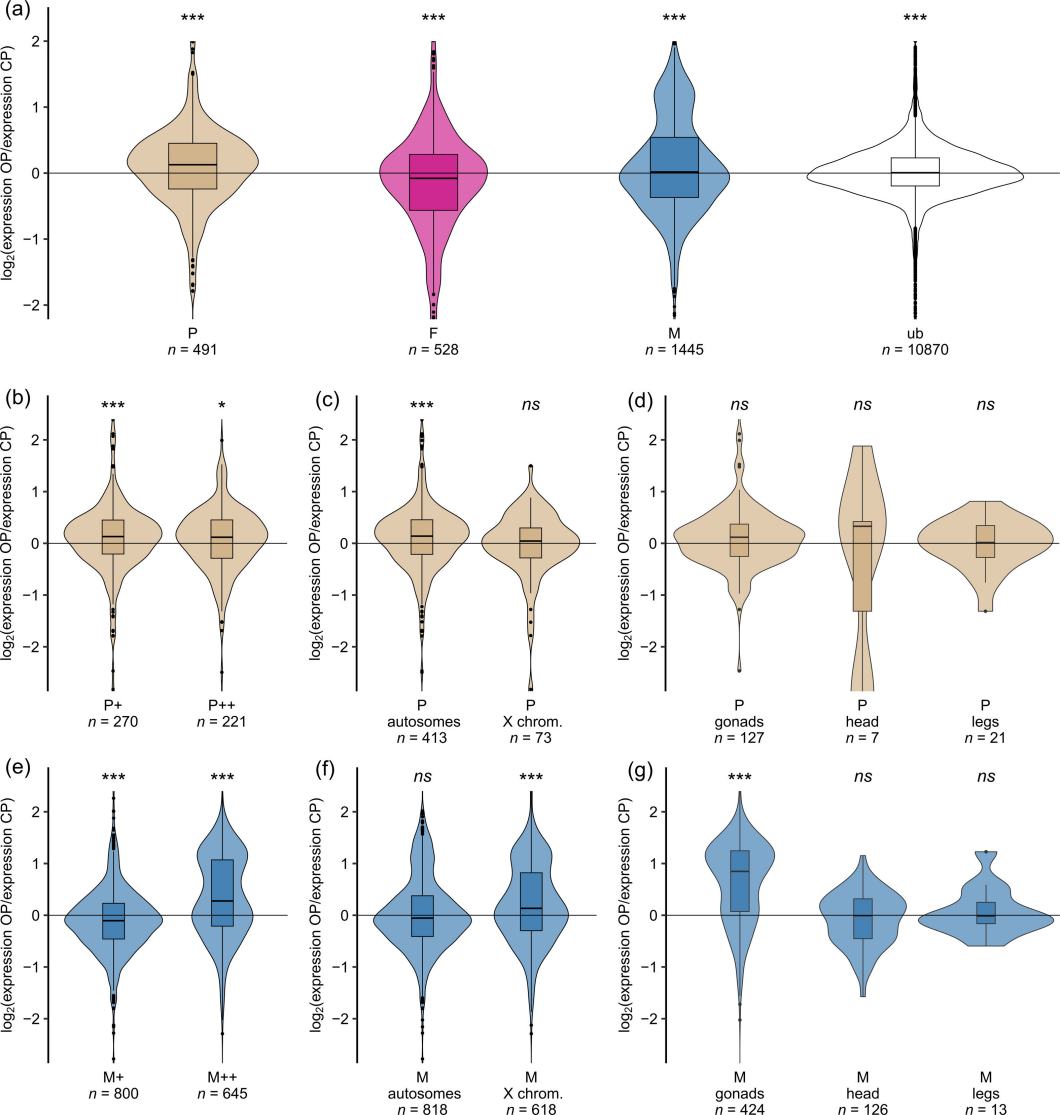
Log_2_-ratio of OP-to-CP gene expression in males of the pea aphid for different categories of genes. OP-to-CP expression ratio as a function of: (*a*) morph-biased status of genes, (*b, e*) degree of morph-bias (+: biased only or ++: limited), (*c, f*) chromosomal location of genes, and (*d, g*) morph- and tissue-biased. status of genes. Colours distinguish different gene expression categories (same as in figure 2).

Our analyses revealed that OP males overexpressed parthenogenetic female-biased genes in comparison with CP males (*Mdn* = 0.127, *p* < 10^–4^, figure 3). Moreover, despite the low median OP-to-CP expression ratio for male-biased genes (*Mdn* = 0.017, *p* = 0.0001), we observed a bimodal density distribution of male-biased gene expression, with a secondary peak for expression ratio around *ca* 1.3 (figure 3) that corresponded to a subset of male-biased genes overexpressed by OP males. Note that while the shift in parthenogenetic female-biased genes was also detected when gene expression classes were determined using clover instead of the alfalfa outgroup, the bimodal distribution was not observed (electronic supplementary material S2). No substantial shift was observed for sexual female-biased and unbiased genes, although the effect was always significant owing to large sample size (figures 3 and 4*a*). The unbiased genes were again distinguished from the morph-biased genes by their much lower variance.

We next wanted to understand which gene classes contributed to the increased expression of parthenogenetic female-biased genes and the increased expression of a subset of male-biased genes in OP males. Interestingly, the increased expression of parthenogenetic female-biased genes by OP males was common to most of the OP–CP pairwise comparisons (electronic supplementary material S10), demonstrating the consistency of this pattern. Both parthenogenetic female-biased only genes (P+, *Mdn* = 0.130, *p* = 0.0005) and parthenogenetic female-limited genes (P++, *Mdn* = 0.117, *p* = 0.017) were significantly more expressed by OP males (figure 4*b*). Contrastingly, only autosomal parthenogenetic female-biased genes were more expressed by OP males (*Mdn* = 0.139, *p* < 10^–5^), with X-linked genes showing no effect (*p* = 0.81, figure 4*c*). Finally, the sub-classification of parthenogenetic female-biased genes did not reveal any different contribution depending on the tissue (figure 4*d*).

Our analyses also revealed that the secondary peak observed for the class of male-biased genes was mostly owing to male-limited genes. Indeed, OP males slightly underexpressed male-biased only genes (M+, *Mdn* = −0.105, *p* < 10^–6^) but clearly overexpressed male-limited genes (M++, *Mdn* = 0.276, *p* < 10^–15^) relative to CP males (figure 4*e*). Male-biased genes assigned to the X chromosome were overexpressed by OP males (*Mdn* = 0.133, *p* < 10^–9^) while autosomal male-biased genes were not (*p* = 0.67, figure 4*f*). Among all male-biased genes with tissue bias, those that were testis-biased were more expressed by OP males than CP males (*Mdn* = 0.847, *p* < 10^–15^, figure 4*g*). For comparison, the same type of analysis (separating genes by chromosome, tissue or expression type) was carried out for sexual female-biased genes, but no strong trend was found (see electronic supplementary material S7G-I). Analyses by chromosome type can be found in electronic supplementary materials S11–S12.

In summary, OP males overexpressed parthenogenetic female-biased genes, as expected under the relaxation of sexual conflict following sex loss. Surprisingly, OP males overexpressed genes that are more specifically expressed in the testes (which additionally tended to be male-limited and more frequent on the X chromosome [19]).

## 4. Discussion

Here, we investigated how the relaxation of sexual conflict in lineages that have lost sex might affect the evolution of gene expression at the whole-genome scale, by comparing gene expression patterns in sexual (CP) and asexual (OP) lineages of the pea aphid. We predicted a shift in gene expression towards the parthenogenetic female optimum in asexual (OP) lineages. Consistent with this prediction, we found that OP males, but not OP parthenogenetic females, tended to express genes normally overexpressed in parthenogenetic females at higher levels than CP males. Unexpectedly, OP males also overexpressed testis genes, and OP parthenogenetic females overexpressed genes that are normally overrepresented in sexual female ovaries. In addition to reduced sexual conflict, the absence of recombination in OP genomes should result in an independent evolution of lineages, leading to a greater divergence of expression among OP relative to CP lineages. However, we did not observe such an effect.

### (a) Morph-biased but not unbiased genes are affected by the transition to permanent asexuality

Analyses of the shift in gene expression associated with the transition to permanent asexuality revealed contrasting results for the different classes of genes (i.e. unbiased and morph-biased). Genes with unbiased expression showed little change in expression between OP and CP lineages, as indicated by an OP-to-CP expression ratio close to 0 and its low variance. This class was also underrepresented among the genes identified as DE between OP and CP males (electronic supplemental material, text 1). The unbiased gene class is likely to contain a high proportion of essential genes or genes with pleiotropic effects that are therefore under strong stabilizing selection for optimal expression and would be little affected by a change in the selective regime, i.e. the disappearance of sexual conflict [3,41]. In contrast, several of the classes of genes defined as morph-biased (either male-, sexual female- or parthenogenetic female-biased) shifted expression between reproductive modes, and some of these classes were also overrepresented in DE genes (see electronic supplementary material, text 1). Sex-biased genes are known to be labile in their expression [42]. They are often thought to have functions related to sexual dimorphism and reproduction and may be involved in past or ongoing intra- or inter-locus sexual conflicts. Therefore, the ongoing arms race between the sexes could explain the high rate of change in expression or sequence generally observed (e.g. [43,44]). These genes would be anticipated to be particularly responsive to a change in the selective regime, namely the disappearance of sexual conflict following sex loss, as we observed here. However, another non-exclusive hypothesis is that sex-biased genes correspond to genes that were once dispensable (their dispensability allowed them to acquire sex-biased expression), so that their expression (and sequences) could still evolve rapidly at low fitness cost [41]. In contrast, differences in tissue allometry (which can account for a significant proportion of the DE genes between the sexes, e.g. [45]) are unlikely to contribute significantly to the observed effects here, as comparisons (OP-to-CP ratio) were made between the same morphs (OP vs CP parthenogenetic females, OP vs CP males). A differential adaption to laboratory conditions between OP and CP lineages can also be ruled out (see the arguments developed in electronic supplementary material, text 2).

### (b) OP males overexpress genes normally overexpressed in parthenogenetic females

OP males overexpressed parthenogenetic female-biased genes compared with CP males, as shown by the OP-to-CP gene expression ratio, a trend observed regardless of the type of expression (biased or limited) and of the way gene expression classes were determined (using the alfalfa outgroup or a fraction of the clover dataset; see electronic supplementary material S2). This is consistent with the prediction of a feminization of male transcriptome under asexuality, with optimal fitness for parthenogenetic females favoured over males, which are no longer under selection. Such feminization of the male transcriptome has also been observed following relaxation of selective pressure on males, resulting from an artificial transition from polygyny to monogamy in *D. melanogaster* [10]. Interestingly, OP pea aphid males also showed a slight decrease in reproductive performance compared to CP males, suggesting relaxed selection on male traits [26]. These results thus show that the effects of relaxed selection on OP males (including the ones resulting from relaxed sexual conflict) can be detected across various phenotypic dimensions. In contrast to our prediction, OP parthenogenetic females did not express parthenogenetic female-biased genes at higher levels compared to CP parthenogenetic females. A hypothesis for this result could be that parthenogenetic females (whether OP or CP) are already at (or close to) their expression optima because parthenogenetic females are the most frequent morph in both OP and CP lineages (representing *ca* 14 out of the *ca* 15 annual generations, even in CP lineages). Consequently, our analyses may not be powerful enough to detect the subtle effect (if any) on parthenogenetic females.

### (c) Asexual morphs overexpress genes primarily expressed in the gonads of sexual morphs

Surprisingly, males from OP lineages express testis genes more than CP males, which goes against our expectations (see also [46]). This pattern (which was nevertheless not observed when clover was used to define gene expression classes; electronic supplementary material S2) is unexpected because OP males are thought to have evolved in a context of relaxed selection. Similarly, OP parthenogenetic females unexpectedly overexpressed sexual female-biased genes normally expressed in sexual female ovaries compared with CP parthenogenetic females. This is surprising because both female morphs have specialized phenotypes (viviparous reproduction without meiosis vs oviparous reproduction with meiosis and mating), making it difficult to understand how the specialized genes of one morph could be re-used by the other. Remarkably, this trend of gonadal gene overexpression by asexual lineages was observed across all lineages, indicating that it is a general pattern.

Most studies (including our own) that have examined how changes in selection regimes during reproduction affect the expression of sex-specific genes have revealed unexpected results. In asexual *Timema* species, parthenogenetic females underexpress sexual female-biased genes but overexpress male-biased genes [13], whereas no change occurs in asexual *Artemia* [14]. Unexpectedly, enforced monogamy in the polygynous *D. pseudoobscura* tends to masculinize the female transcriptome [12]. Only one [10] of the two studies [10,11] on *D. melanogaster* fits the predictions, with a feminization of male and female transcriptomes under enforced monogamy. Various hypotheses have been put forward to explain these unexpected patterns. In particular, in *Timema* and *Artemia*, the loss of sex is confounded with the emergence of a new reproductive morph, the parthenogenetic female morph. The fitness optimum for this newly emerged morph should not necessarily be similar to that of sexual female (the former no longer needs to mate and is able to produce diploid eggs through modified cellular processes), which could raise different expectations. Parker *et al*. [13] therefore argued that the observed masculinization of the transcriptome of asexual females is not directly owing to the release of sexual conflict in asexual lineages, but rather to a change in the optimal female phenotype. This hypothesis is further supported by the observation of convergent changes in gene expression across five independent transitions to asexuality in *Timema* [47]. However, in the pea aphid, the difference in optima for parthenogenetic females in OP and CP lineages is unlikely to explain changes in expression because parthenogenetic females are originally part of the CP life cycle. In contrast, a recurring problem in all studies (including ours) that examine expression changes associated with sex loss is the confounding effect of geography (or ecological niche). Indeed, asexuals generally differ from their sexual relatives in this respect (e.g. *Timema* [48], *Artemia* [49]; see also [50]). This implies that changes in gene expression can result not only from the loss of sex but also from local adaptation. On the other hand, geographical isolation is essential for studying the evolutionary consequences of changes in reproductive strategies because it limits the genetic mixing of populations with different reproductive strategies (as in aphids). Consequently, these confounding effects are difficult to avoid when studying wild populations, which nevertheless provide a complementary perspective to experimentally induced changes in mating systems (usually over a smaller number of generations, e.g. [10–12]). In the specific case of aphids, the genes involved in local adaptation should be more likely to have a more general functions (e.g. adaptation to thermal stress or pesticides) and we see no obvious reason why sex-biased and gonadal genes would be primarily affected.

Several hypotheses, whether adaptive or not, can be put forward to explain the overexpression of gonadal genes in OP males and OP parthenogenetic females. A first non-adaptive hypothesis is that these expression profiles in OP lineages may have been inherited from a common (and relatively recent) OP ancestor that, by chance, already had a slightly atypical expression profile. This would explain the consistency of the overexpression of sexual female ovary genes (in parthenogenetic females) and testis genes (in males) across all four OP lineages. Population history can indeed be a major source of variation in gene expression, as shown in asexual and sexual populations of freshwater snails [51]. However, our analyses of the genetic relationships between the four OP and four CP lineages based on >10 000 SNPs (current study) and a larger panel of 23 OP and 76 CP lineages using a lower number of markers (15 microsatellite loci [26]) contradict this hypothesis. Although the SNP tree grouped the four OP lineages with excellent bootstrap support, one OP lineage (OP1) was the closest to the CP lineages. On the 15-microsatellite tree, the OP1 lineage is closer to CP lineages than to OP lineages, suggesting possible independent transitions to permanent asexuality. Furthermore, although the genetic distances among the OP lineages are marginally lower than those between the CP lineages, the values are quite similar, suggesting that the OP lineages have been evolving for a comparable length of time to the CP lineages. Recent common ancestry is therefore unlikely to explain the peculiar expression of sexual gonad genes in OP lineages. A second non-adaptive hypothesis for the shift in expression of genes from the sexual gonads could be that the dosage compensation system has been disrupted in OP lineages. Indeed, dosage compensation would be expected to decay because the lack of reproductive opportunities for OP males leads to relaxed selection on males. However, we found no major change in dosage compensation in OP compared to CP lineages, as assessed by comparing the male-to-female expression ratio for autosomal and X-linked genes between OP and CP lineages (see electronic supplementary material, text 1). A third hypothesis could be that in OP lineages, the genetic programme that normally controls gonadal genes in CP sexual morphs is dysregulated. Indeed, the absence of sexual females in OP lineages and the presumed lack of reproductive opportunity for OP males means that selection is no longer acting on these morphs, and in particular on the genes that are specific to these morphs. Small changes in one or more upstream regulatory genes may be sufficient to affect a large number of downstream genes. Evolutionary changes in regulatory regions are frequent [52], with direct evidence found in animals [53], fungi [54] and plants [55]. Alternatively, sex-biased genes, especially those restricted to gonads, evolve rapidly at the protein level (e.g. [42,56,57]). Hence, genes initially linked to sexual reproduction owing to their predominant expression in sexual morph gonads within CP lineages may have evolved functional modifications that confer greater advantages to parthenogenetic females. A detailed study of these sexual female ovary genes is required to elucidate their precise functions, yet the substantial number of genes with unknown function in the pea aphid poses a significant challenge.

### (d) Non-recombining OP lineages do not show increased divergence in gene expression

The loss of sex causes the genome to stop recombining, allowing OP lineages to evolve independently, which should increase expression divergence between them. The lack of recombination also reduces the efficiency of selection, resulting in deleterious mutation accumulation with potential snowballing effects on downstream genes. Few studies have examined the evolution of gene expression within populations that differ in recombination rate. Theoretical models predict that lack of recombination would eventually lead to haploidization of expression, with one of the two alleles at a diploid locus contributing the majority of expression via an enhancer runaway divergence process [58]. However, no prediction was made regarding the expected expression divergence between asexual lineages. Empirical evidence for gene expression divergence in apomictic (asexual) plant lineages is provided by [59], but no comparisons were made with sexual counterparts. Ma *et al*. [60] investigated the allele-specific expression in non-recombining regions of the sex chromosomes of a fungus and showed evidence for a relationship between expression divergence between alleles and allele degeneration. To our knowledge, our study is the first to compare gene expression divergence within different reproductive modes. Contrary to our expectations of increased divergence between OP lineages, we observed no effect of the reproductive mode on transcriptomic distances, corrected or not for genetic distances. Mathematical modelling may be necessary to verify the accuracy of our predictions regarding the evolution of gene expression in the absence of recombination. Purifying selection may also play a role, wherein OP lineages exhibiting lesser expression divergence experience enhanced fitness, consequently limiting the divergence among OP lineages.

## 5. Conclusion

Our study highlights the evolutionary implications of the loss of sexual reproduction, which results in the cessation of intra-locus sexual conflict and recombination. We found substantial changes in the expression of morph-biased genes, but not always in the expected direction. This suggests that processes other than the reduction of sexual conflict may influence the evolution of gene expression in asexual lineages. Our results should also be interpreted with some caution owing to the confounding effects of geography and sex loss inherent to many sexual–asexual complexes. It would also be interesting to investigate whether an ‘enhancer runaway divergence’ process occurs in OP lineages owing to the accumulation of deleterious mutations and the lack of recombination, and whether this process contributes to the expression changes. In addition, further investigation at the DNA sequence level would be valuable, for example, to test whether genes typically restricted to sexual female ovaries accumulate deleterious mutations in OP lineages owing to the lack of selection, or conversely, exhibit signs of positive selection, thus unveiling adaptive changes. Finally, more theoretical studies are needed to better capture the importance and role of sexual conflicts on gene expression evolution from empirical datasets.

## Data Availability

Raw reads have been deposited in the SRA on NCBI, under the BioProject accession number PRJNA1024923. The data (read counts and genotype data) and scripts have been deposited on Zenodo [61] and the remaining data are also included online as electronic supplementary material [62].

## References

[1] Bonduriansky R, Chenoweth SF. 2009 Intralocus sexual conflict. Trends Ecol. Evol. **24**, 280–288. (10.1016/j.tree.2008.12.005) 19307043

[2] Lande R. 1980 Sexual dimorphism, sexual selection, and adaptation in polygenic characters. Evolution **34**, 292. (10.2307/2407393) 28563426

[3] Mank JE, Hultin‐Rosenberg L, Zwahlen M, Ellegren H. 2008 Pleiotropic constraint hampers the resolution of sexual antagonism in vertebrate gene expression. Am. Nat. **171**, 35–43. (10.1086/523954) 18171149

[4] Griffin RM, Dean R, Grace JL, Ryden P, Friberg U. 2013 The shared genome is a pervasive constraint on the evolution of sex-biased gene expression. Mol. Biol. Evol. **30**, 2168–2176. (10.1093/molbev/mst121) 23813981

[5] Tosto NM, Beasley ER, Wong BBM, Mank JE, Flanagan SP. 2023 The roles of sexual selection and sexual conflict in shaping patterns of genome and transcriptome variation. Nat. Ecol. Evol. **7**, 981–993. (10.1038/s41559-023-02019-7) 36959239

[6] Cheng C, Kirkpatrick M. 2016 Sex-specific selection and sex-biased gene expression in humans and flies. PLOS Genet. **12**, e1006170. (10.1371/journal.pgen.1006170) 27658217 PMC5033347

[7] Chippindale AK, Gibson JR, Rice WR. 2001 Negative genetic correlation for adult fitness between sexes reveals ontogenetic conflict in Drosophila. Proc. Natl. Acad. Sci. **98**, 1671–1675. (10.1073/pnas.98.4.1671) 11172009 PMC29315

[8] Innocenti P, Morrow EH. 2010 The sexually antagonistic genes of Drosophila melanogaster. PLoS Biol. **8**, e1000335. (10.1371/journal.pbio.1000335) 20305719 PMC2838750

[9] Holland B, Rice WR. 1999 Experimental removal of sexual selection reverses intersexual antagonistic coevolution and removes a reproductive load. Proc. Natl. Acad. Sci. **96**, 5083–5088. (10.1073/pnas.96.9.5083) 10220422 PMC21820

[10] Hollis B, Houle D, Yan Z, Kawecki TJ, Keller L. 2014 Evolution under monogamy feminizes gene expression in Drosophila melanogaster. Nat. Commun. **5**, 3482. (10.1038/ncomms4482) 24637641

[11] Mishra P, Rundle HD, Agrawal AF. 2024 The evolution of sexual dimorphism in gene expression in response to a manipulation of mate competition. Evolution **78**, 746–757. (10.1093/evolut/qpae004) 38270064

[12] Veltsos P, Fang Y, Cossins AR, Snook RR, Ritchie MG. 2017 Mating system manipulation and the evolution of sex-biased gene expression in Drosophila. Nat. Commun. **8**, 2072. (10.1038/s41467-017-02232-6) 29233985 PMC5727229

[13] Parker DJ, Bast J, Jalvingh K, Dumas Z, Robinson-Rechavi M, Schwander T. 2019 Sex-biased gene expression is repeatedly masculinized in asexual females. Nat. Commun. **10**, 4638. (10.1038/s41467-019-12659-8) 31604947 PMC6789136

[14] Huylmans AK, Macon A, Hontoria F, Vicoso B. 2021 Transitions to asexuality and evolution of gene expression in Artemia brine shrimp. Proc. R. Soc. B **288**, 20211720. (10.1098/rspb.2021.1720) PMC8456138 34547909

[15] Maynard Smith J. 1978 The evolution of sex. Cambridge, UK: Cambridge University Press.

[16] Dixon A. 1998 Aphid ecology, 2nd edn. London, UK: Chapman & Hall.

[17] Davis GK. 2012 Cyclical parthenogenesis and vviparity in aphids as evolutionary novelties. J. Exp. Zool. Part B **318**, 448–459. (10.1002/jez.b.22441) 22644631

[18] Wilson ACC, Sunnucks P, Hales DF. 1997 Random loss of X chromosome at male determination in an aphid, Sitobion near fragariae, detected using an X-linked polymorphic microsatellite marker. Genet. Res. **69**, 233–236. (10.1017/S0016672397002747)

[19] Jaquiéry J *et al*. 2022 Masculinization of the X-chromosome in aphid soma and gonads. Peer Community J. **2**. (10.24072/pcjournal.166)

[20] Jaquiéry J *et al*. 2013 Masculinization of the X chromosome in the pea aphid. PLoS Genet. **9**, e1003690. (10.1371/journal.pgen.1003690) 23950732 PMC3738461

[21] Gautam DC, Crema R, Pagliai AMB. 1993 Cytogenetic mechanisms in aphids. Boll. Di Zool. **60**, 233–244. (10.1080/11250009309355818)

[22] Simon JC, Rispe C, Sunnucks P. 2002 Ecology and evolution of sex in aphids. Trends Ecol. Evol. **17**, 34–39. (10.1016/s0169-5347(01)02331-x)

[23] Simon JC, Stoeckel S, Tagu D. 2010 Evolutionary and functional insights into reproductive strategies of aphids. Comptes Rendus Biol. **333**, 488–496. (10.1016/j.crvi.2010.03.003) 20541160

[24] Simon JC, Baumann S, Sunnucks P, Hebert PDN, Pierre JS, Le Gallic JF, Dedryver CA. 1999 Reproductive mode and population genetic structure of the cereal aphid Sitobion avenae studied using phenotypic and microsatellite markers. Mol. Ecol. **8**, 531–545. (10.1046/j.1365-294x.1999.00583.x) 10327655

[25] Vorburger C, Lancaster M, Sunnucks P. 2003 Environmentally related patterns of reproductive modes in the aphid Myzus persicae and the predominance of two ‘superclones’ in Victoria, Australia. Mol. Ecol. **12**, 3493–3504. (10.1046/j.1365-294x.2003.01998.x) 14629364

[26] Defendini H, Rimbault M, Mahéo F, Cloteau R, Denis G, Mieuzet L, Outreman Y, Simon JC, Jaquiéry J. 2023 Evolutionary consequences of loss of sexual reproduction on male-related traits in parthenogenetic lineages of the pea aphid. Mol. Ecol. **32**, 3672–3685. (10.1111/mec.16961) 37143321

[27] Le Trionnaire G *et al*. 2009 Transcriptomic and proteomic analyses of seasonal photoperiodism in the pea aphid. BMC Genom. **10**, 456. (10.1186/1471-2164-10-456) PMC2763885 19788735

[28] Mathers TC, Wouters RHM, Mugford ST, Swarbreck D, van Oosterhout C, Hogenhout SA. 2021 Chromosome-scale genome assemblies of aphids reveal extensively rearranged autosomes and long-term conservation of the X chromosome. Mol. Biol. Evol. **38**, 856–875. (10.1093/molbev/msaa246) 32966576 PMC7947777

[29] Legeai F *et al*. 2010 AphidBase: a centralized bioinformatic resource for annotation of the pea aphid genome. Insect Mol. Biol. **19**, 5–12. (10.1111/j.1365-2583.2009.00930.x) 20482635 PMC4372297

[30] Dobin A, Davis CA, Schlesinger F, Drenkow J, Zaleski C, Jha S, Batut P, Chaisson M, Gingeras TR. 2013 STAR: ultrafast universal RNA-seq aligner. Bioinformatics **29**, 15–21. (10.1093/bioinformatics/bts635) 23104886 PMC3530905

[31] Liao Y, Smyth GK, Shi W. 2014 featureCounts: an efficient general purpose program for assigning sequence reads to genomic features. Bioinformatics **30**, 923–930. (10.1093/bioinformatics/btt656) 24227677

[32] Chen Y, Lun ATL, Smyth GK. 2016 From reads to genes to pathways: differential expression analysis of RNA-Seq experiments using Rsubread and the edgeR quasi-likelihood pipeline. F1000Research **5**, 1438. (10.12688/f1000research.8987.2) 27508061 PMC4934518

[33] R Core Team. 2023 R: A language and environment for statistical computing. Vienna, Austria: R Foundation for Statistical Computing.

[34] Li H. 2011 A statistical framework for SNP calling, mutation discovery, association mapping and population genetical parameter estimation from sequencing data. Bioinformatics **27**, 2987–2993. (10.1093/bioinformatics/btr509) 21903627 PMC3198575

[35] Nei M. 1978 Estimation of average heterozygosity and genetic distance from a small number of individuals. Genetics **89**, 583–590. (10.1093/genetics/89.3.583) 17248844 PMC1213855

[36] Kamvar ZN, Tabima JF, Grünwald NJ. 2014 Poppr : an R package for genetic analysis of populations with clonal, partially clonal, and/or sexual reproduction. PeerJ **2**, e281. (10.7717/peerj.281) 24688859 PMC3961149

[37] Dray S, Dufour AB. 2007 The ade4 Package: Implementing the duality diagram for ecologists. J. Stat. Softw. **22**, 1–20. (10.18637/jss.v022.i04)

[38] Oksanen J, Simpson G, Blanchet F, Kindt R, Legendre P, Minchin P, O’Hara RB. 2022 See https://github.com/vegandevs/vegan.

[39] Benjamini Y, Hochberg Y. 1995 Controlling the false discovery rate: a practical and powerful approach to multiple testing. J. R. Stat. Soc. **57**, 289–300. (10.1111/j.2517-6161.1995.tb02031.x)

[40] Lewis M. 2021 glmmSeq: General linear mixed models for gene-level differential expression. See https://cran.r-project.org/web/packages/glmmSeq.

[41] Mank JE, Ellegren H. 2009 Are sex-biased genes more dispensable? Biol. Lett. **5**, 409–412. (10.1098/rsbl.2008.0732) 19433612 PMC2679909

[42] Ellegren H, Parsch J. 2007 The evolution of sex-biased genes and sex-biased gene expression. Nat. Rev. Genet. **8**, 689–698. (10.1038/nrg2167) 17680007

[43] Grath S, Parsch J. 2016 Sex-biased gene expression. Annu. Rev. Genet. **50**, 29–44. (10.1146/annurev-genet-120215-035429) 27574843

[44] Wright AE, Fumagalli M, Cooney CR, Bloch NI, Vieira FG, Buechel SD, Kolm N, Mank JE. 2018 Male-biased gene expression resolves sexual conflict through the evolution of sex-specific genetic architecture. Evol. Lett. **2**, 52–61. (10.1002/evl3.39) 30283664 PMC6089503

[45] Darolti I, Mank JE. 2023 Sex-biased gene expression at single-cell resolution: cause and consequence of sexual dimorphism. Evol. Lett. **7**, 148–156. (10.1093/evlett/qrad013) 37251587 PMC10210449

[46] Dean R, Hammer C, Higham V, Dowling DK. 2018 Masculinization of gene expression is associated with male quality in Drosophila melanogaster. Evol. Int. J. Org. Evol. **72**, 2736–2748. (10.1111/evo.13618) 30382578

[47] Parker DJ, Bast J, Jalvingh K, Dumas Z, Robinson-Rechavi M, Schwander T. 2019 Repeated evolution of asexuality involves convergent gene expression changes. Mol. Biol. Evol. **36**, 350–364. (10.1093/molbev/msy217) 30445505 PMC6404633

[48] Larose C, Parker DJ, Schwander T. 2018 Fundamental and realized feeding niche breadths of sexual and asexual stick insects. Proc. R. Soc. B. **285**, 20181805. (10.1098/rspb.2018.1805) PMC6283937 30487310

[49] Eimanifar A, Stappen G, Wink M. 2015 Geographical distribution and evolutionary divergence times of Asian populations of the brine shrimp Artemia (Crustacea, Anostraca). Zool. J. Linn. Soc. **174**, 447–458. (10.1111/zoj.12242)

[50] Neiman M, Meirmans PG, Schwander T, Meirmans S. 2018 Sex in the wild: how and why field-based studies contribute to solving the problem of sex. Evol. Int. J. Org. Evol. **72**, 1194–1203. (10.1111/evo.13485) 29645091

[51] McElroy KE, Bankers L, Soper D, Hehman G, Boore JL, Logsdon JM, Neiman M. 2022 Patterns of gene expression in ovaries of sexual vs. asexual lineages of a freshwater snail. Front. Ecol. Evol. **10**, 845640. (10.3389/fevo.2022.845640)

[52] Johnson AD. 2017 The rewiring of transcription circuits in evolution. Curr. Opin. Genet. Dev. **47**, 121–127. (10.1016/j.gde.2017.09.004) 29120735 PMC6901287

[53] Erkenbrack EM, Davidson EH. 2015 Evolutionary rewiring of gene regulatory network linkages at divergence of the echinoid subclasses. Proc. Natl Acad. Sci. USA **112**, E4075–84. (10.1073/pnas.1509845112) 26170318 PMC4522742

[54] Wilinski D, Buter N, Klocko AD, Lapointe CP, Selker EU, Gasch AP, Wickens M. 2017 Recurrent rewiring and emergence of RNA regulatory networks. Proc. Natl Acad. Sci. USA **114**, E2816–E2825. (10.1073/pnas.1617777114) 28320951 PMC5389312

[55] Muiño JM, de Bruijn S, Pajoro A, Geuten K, Vingron M, Angenent GC, Kaufmann K. 2016 Evolution of DNA-binding sites of a floral master regulatory transcription factor. Mol. Biol. Evol. **33**, 185–200. (10.1093/molbev/msv210) 26429922 PMC4693976

[56] Meisel RP. 2011 Towards a more nuanced understanding of the relationship between sex-biased gene expression and rates of protein-coding sequence evolution. Mol. Biol. Evol. **28**, 1893–1900. (10.1093/molbev/msr010) 21239389 PMC3098513

[57] Whittle CA, Johannesson H. 2013 Evolutionary dynamics of sex-biased genes in a hermaphrodite fungus. Mol. Biol. Evol. **30**, 2435–2446. (10.1093/molbev/mst143) 23966547

[58] Fyon F, Lenormand T. 2018 Cis-regulator runaway and divergence in asexuals. Evolution **72**, 426–439. (10.1111/evo.13424) 29331019

[59] Ferreira de Carvalho J, Oplaat C, Pappas N, Derks M, de Ridder D, Verhoeven KJF. 2016 Heritable gene expression differences between apomictic clone members in Taraxacum officinale: insights into early stages of evolutionary divergence in asexual plants. BMC Genom. **17**, 203. (10.1186/s12864-016-2524-6) PMC4782324 26956152

[60] Ma WJ, Carpentier F, Giraud T, Hood ME. 2020 Differential gene expression between fungal mating types is associated with sequence degeneration. Genome Biol. Evol. **12**, 243–258. (10.1093/gbe/evaa028) 32058544 PMC7150583

[61] Defendini H, Jaquiéry J. 2025 Datasets and script for: The release of sexual conflict after sex loss is associated with evolutionary changes in gene expression. Zenodo (10.5281/zenodo.14512762) PMC11775605 39876718

[62] Defendini H, Prunier-Leterme N, Robin S, Lameiras S, Baulande S, Simon JC *et al*. 2025 Supplementary material from: The release of sexual conflict after sex loss is associated with evolutionary changes in gene expression. Figshare (10.6084/m9.figshare.c.7634177) PMC11775605 39876718

